# Supplementary material for: Anisotropic thermal conductivity of antigorite along slab subduction impacts seismicity of intermediate-depth earthquakes
Source: Nat Commun. 2024 Jun 18;15:5198. doi: 10.1038/s41467-024-49418-3 (PMC11189502; doi:10.1038/s41467-024-49418-3)
Supplement: Supplementary file 1 — Supplementary Information [file 41467_2024_49418_MOESM1_ESM.pdf]

Supplementary Information for

Anisotropic thermal conductivity of antigorite along slab subduction impacts seismicity of intermediate-depth earthquakes

by Chien et al.

Note S1. Physical Model

We designed a 2D numerical model to quantify the effect of antigorite's thermal conductivity anisotropy on the slab's thermal evolution. To perform the calculation, we employed a self-written MATLAB script. The slab was represented as a rectangular object with a thickness H_{slab}, and a length $L_{slab} = 5H_{slab}$ (Fig. S5). The slab thickness H_{slab} was computed with the analytical solution reported by Ref [1]:

$$H_{slab} = 2.32\sqrt{\kappa t} \quad (1)$$

where t is the slab age, here assumed as $t \approx 2.52 \times 10^{15}\ s$ ($80\ Myrs$), and κ is the thermal diffusivity set to $\kappa = 1 \times 10^{-6}\ m^2\ s^{-1}$. The resulting slab was $120\ km$ thick, and $600\ km$ long. The value of $80\ Myrs$ was chosen because it is considered as the upper limit for the slab thickening, representing a good proxy for a cold old slab[2].

The cold slab subducted vertically into the mantle, i.e., $\theta_{dip} = 90°$, and it was progressively heated by the hot mantle (see Fig. S6). The slab was composed by three lithologies layered as follows: (i) a meta-basaltic crust on top ($7\ km$ thick)[3], (ii) a layer of hydrated harzburgitic lithosphere in the middle ($3\ km$ thick)[4], and (iii) a dry harzburgitic lithosphere keel at the bottom ($110\ km$ thick)[5].

The initial slab temperature profile at the trench was computed using the half-space cooling equation[1]:

$$T_x = T_0 + \operatorname{erf}\left(\frac{x}{2\sqrt{\kappa t}}\right) * (T_m - T_0) \quad (2)$$

In this equation, we assumed a surface temperature of $T_0 = 300\ K,$ and an ambient mantle temperature of $T_m = 1600\ K.$ The parameters κ and t are the same of eq. 1, whereas x (m) represents the spatial coordinate. This equation produced a 1D temperature profile along the slab thickness, and the solution was truncated at $120\ km$ of depth. The temperature transect at the trench was then extended to the rest of the slab (Fig. S5).

Slab subduction was simulated from $120\ km$ to $230\ km$ depth by imposing a constant sinking velocity of $v_{sink} = 5\ cm\ yr^{-1}$. The entry depth of the slab in the mantle corresponds to the depth of the bottom part of the lithosphere, and it was chosen in order to avoid to simulate complex interactions between the slab and the overriding plate at the trench. The depth of $230\ km$ corresponds to the depth of antigorite's breakdown[6].

The dominant heat transport mechanism inside the subducting slab is diffusion. The heat diffusion is described by the equation:

$$\rho Cp\left(\frac{\partial T}{\partial t}\right) = -\nabla \cdot (-\Lambda \nabla T) \tag{3}$$

where $(\partial T/\partial t)$ indicates the temperature evolution over time ($K\ s^{-1}$), ∇T is the temperature gradient ($K\ m^{-1}$) in 3D space $(x,\ y,\ z)$, $\nabla\cdot$ is the divergence in 3D space $(\partial \mathfrak{f}_x/\partial x) + (\partial \mathfrak{f}_y/\partial y) + (\partial \mathfrak{f}_z/\partial z)$, ρ $(kg\ m^{-3})$ is the density, Cp $(J\ kg^{-1}K^{-1})$ is the specific heat capacity and Λ $(W\ m^{-1}K^{-1})$ is the thermal conductivity. In 2D the heat diffusion equation becomes:

$$\rho Cp\left(\frac{\partial T}{\partial t}\right) = \frac{\partial}{\partial x}\left(\Lambda_H \frac{\partial T}{\partial \mathrm{x}}\right) + \frac{\partial}{\partial y}\left(\Lambda_V \frac{\partial T}{\partial \mathrm{y}}\right) \tag{4}$$

Given the model geometry of the sinking slab, the x-direction represents the horizontal axis along the slab thickness, whereas the y-direction represents the vertical axis along the slab length (Fig. S7). To investigate the anisotropy of antigorite's thermal conductivity we distinguished the horizontal Λ_H and vertical Λ_V components along the corresponding axis. Antigorite was the only mineral in the model with $\Lambda_H \neq \Lambda_V$, whereas olivine $\Lambda_H = \Lambda_V$.

During the slab descent, the pressure and the temperature conditions were increased with depth. Pressure was increased by using the Preliminary Reference Earth Model (PREM) from Ref [7] (Fig. S8). The profile was built using a spline function with a 2nd order polynomial and 10 intervals (Table S1 for the coefficients).

$$P(D) = cD^2 + bD + a \tag{5}$$

The depth-dependent ambient mantle temperature was reproduced using the adiabatic temperature gradients ΔT $(K\ km^{-1})$ reported by Ref [8] (Fig. S8).

$$T(D+1) = T(D) + \Delta D * \Delta T(D) \tag{6}$$

The compression-induced temperature increase ΔT was added to slab temperatures as well as the ambient mantle. The progressive increase of the P-T conditions experienced by the descending of the slab determine the variation of the materials' parameters Λ, ρ, Cp (see Note S3–S5). The governing equations of Λ, ρ, and Cp are reported in Note S3–S5 and Table S2–S3.

Note S2. Numerical Model

To solve the partial differential equation for 2D heat diffusion (eq. 4.) we employed the finite difference (FD) method[9]. The domain was discretized into square cells with a constant grid space of $\Delta x = \Delta y = 500\ m$. The resulting grid mesh was composed by 242×1202 nodes. For the calculations we used the central difference method[9], and the discretized heat diffusion equation reads:

$$\rho_i Cp_i \left(\frac{\Delta T_i}{\Delta t}\right) = \frac{1}{\Delta x^2}\left[\Lambda_B\left(T_{i,j+1} - T_{i,j}\right) - \Lambda_A\left(T_{i,j} - T_{i,j-1}\right)\right] + \frac{1}{\Delta y^2}\left[\Lambda_\beta\left(T_{i+1,j} - T_{i,j}\right) - \Lambda_\alpha\left(T_{i,j} - T_{i-1,j}\right)\right] \quad (7)$$

In this discretization, the temperature variations in a central node T_{ij} is computed considering the four neighbouring nodes: $T_{i-1,j}$ (upper), $T_{i+1,j}$ (lower), $T_{i,j-1}$ (left), and $T_{i,j+1}$ (right). The equation 7 was computed considering density ρ_i and specific heat capacity Cp_i at central node $[i, j]$. Since thermal conductivity is a flux-controlling parameter ($J\ s^{-1}m^{-1}K^{-1}$), it is necessary to employ the conservative discretization in order to avoid artificial energy flux at sharp Λ discontinuities[9]. The two parameter Λ_A , Λ_B represent the horizontal thermal conductivities at the intermediate positions $A\ [i, j - \frac{1}{2}]$ and $B\ [i, j + \frac{1}{2}]$, whereas Λ_α , Λ_β represent the vertical thermal conductivities at the intermediate positions $\alpha\ [i - \frac{1}{2}, j]$ and $\beta\ [\ j + \frac{1}{2}, j]$. The chosen time step Δt was $7.89 \times 10^{10}\ s$, corresponding to $2000\ yrs$. The code was benchmarked for the prescribed Δx and Δt (Note S6).

To form the boundaries of the model, we prescribed four extra stencils of nodes in the four sides of the domain. Each node of the boundary was set to have isothermal conditions for the entire duration of the simulation. At depth above $120\ km$ (i.e., slab entry point in the mantle) the boundaries represented a 500-m-thick layer of ambient mantle. Therefore, the temperature of a given boundary node depended on the relative position of the slab along the adiabatic profile of the mantle (eq. 6). Below $120\ km$ depth, the boundaries were designed to maintain the slab at the initial temperature conditions of the trench. To limit the internal heat diffusion, we set the left and right boundaries of the domain with the same temperature of the edges of the slab. Therefore, the right side of the domain, which represented the top of the

vertically sinking slab, was set to $300\ K$. The left side of domain representing the bottom of the slab was set to the same temperature of the truncated half-space cooling profile. The heat diffusion equation was solved implicitly with the direct solver algorithm '*mldivide*', implemented in Matlab as the operator '\'.

Note S3. Thermal Conductivity Λ

Thermal conductivities of slab minerals are based on TDTR measurements at high pressures and variable temperatures. The thermal conductivities of different mantle phases, as a function of pressure $\Lambda^x(P)$, are expressed with a 4th order polynomial:

$$\Lambda^{Ol}(P) = d_x P^4 + c_x P^3 + b_x P^2 + a_x P + \Lambda_0^{Ol} \quad (8)$$

The subscript 'x' indicates the given mineral phase (e.g., Λ^{Ol} for olivine), the letters from a-d represents the various coefficients, and Λ_0^x stands for the thermal conductivity at ambient pressure. See Table S2 for a summary of all coefficients.

Temperature dependent Λ^x follows the relation reported in the present study and Ref [10,11]:

$$\Lambda^x(T) = \Lambda_{298}^x \left(\frac{T}{298}\right)^{-z_x} \quad (9)$$

where Λ_{298}^x is the thermal conductivity at high-P and room-T $(298\ K)$. The exponent z_x represents the T-dependent coefficient for the given phase.

The aggregate thermal conductivity of each slab lithology was computed as the geometric average among the contributions of its constituent minerals[12]:

$$\Lambda_{rock} = (\Lambda^1)^{\varphi_1} * (\Lambda^2)^{\varphi_2} * \cdots * (\Lambda^n)^{\varphi_n} \quad (10)$$

where Λ^n is the thermal conductivity of the phase n, and φ_n is its volume fraction in the rock. The lithospheric slab was assumed to be made entirely of olivine ($\varphi_{Ol} = 1$), using the lattice thermal conductivity Λ^{Ol} reported by Ref [13]:

$$\Lambda_{LitSlab} = \Lambda_{Crust} = (\Lambda^{Ol})^{\varphi_{Ol}} \quad (11)$$

Such assumption is justified by the argument that thermal conductivities of pyroxene and garnet, the main components of the meta-basaltic crust, are expected to be similar to the olivine[14,15]. In this model, we assumed a completely dry meta-basaltic crust and we concentrated all the hydrous minerals in 3-km-thick layer at the interface between the crust and the lithosphere (Fig. S6), as proposed by Ref [16]. The aggregate thermal conductivity of the hydrous layer Λ_{Hyd} was computed by including the contributions of olivine Λ^{Ol} [13] and antigorite Λ^{Atg} measured in this study:

$$\Lambda_{Hyd} = (\Lambda^{Ol})^{\varphi_{Ol}} * (\Lambda^{Atg})^{\varphi_{Atg}} \quad (12)$$

A 15 $vol\%$ antigorite corresponds to an hydrous layer with $2wt\%$ H_2O[17,18]. To simulate different scenarios of lithosphere hydration, we changed the volume fraction of antigorite φ_{Atg} in each model: $\varphi_{Atg} = 0.1$, 0.3, 0.5, and 1. The 0.3 and 0.5 cases represent pervasive alteration scenarios, which can be found, for example, in the Atlantic oceanic lithosphere[19]. The case of $\varphi_{Atg} = 1$, on the other hand, represents an end-member scenario, which assumes the complete serpentinization of the hydrous layer.

For our present study, we run 10 different models (Figure S9a-c):

I. Dry olivine and no serpentinization (0 $vol\%$ antigorite)

$$\Lambda_H^{HydLayer} = \Lambda^{DryOl} \qquad (13)$$
$$\Lambda_V^{HydLayer} = \Lambda^{DryOl}$$

II. Wet olivine and no serpentinization (0 $vol\%$ antigorite)

$$\Lambda_H^{HydLayer} = \Lambda^{WetOl} \qquad (14)$$
$$\Lambda_V^{HydLayer} = \Lambda^{WetOl}$$

III. Dry olivine and low serpentinization (10 $vol\%$ antigorite). Antigorite oriented with the [010] face horizontally, and the [001] face vertically.

$$\Lambda_H^{HydLayer} = (\Lambda^{Ol})^{0.9} * (\Lambda^{010})^{0.1} \qquad (15)$$
$$\Lambda_V^{HydLayer} = (\Lambda^{Ol})^{0.9} * (\Lambda^{001})^{0.1}$$

IV. Dry olivine and intermediate serpentinization (30 $vol\%$ antigorite). Antigorite oriented with the [010] face horizontally, and the [001] face vertically.

$$\Lambda_H^{HydLayer} = (\Lambda^{Ol})^{0.7} * (\Lambda^{010})^{0.3} \qquad (16)$$
$$\Lambda_V^{HydLayer} = (\Lambda^{Ol})^{0.7} * (\Lambda^{001})^{0.3}$$

V. Dry olivine and high serpentinization (50 $vol\%$ antigorite). Antigorite oriented with the [010] face horizontally, and the [001] face vertically.

$$\Lambda_H^{HydLayer} = (\Lambda^{Ol})^{0.5} * (\Lambda^{010})^{0.5} \qquad (17)$$
$$\Lambda_V^{HydLayer} = (\Lambda^{Ol})^{0.5} * (\Lambda^{001})^{0.5}$$

VI. Complete serpentinization (100 $vol\%$ antigorite). Antigorite oriented with the [010] face horizontally, and the [001] face vertically

$$\Lambda_H^{HydLayer} = \Lambda^{010} \qquad (18)$$
$$\Lambda_V^{HydLayer} = \Lambda^{001}$$

VII. Dry olivine and low serpentinization (10 $vol\%$ antigorite). Antigorite oriented with the [001] face horizontally, and the [010] face vertically.

$$\Lambda_H^{HydLayer} = (\Lambda^{Ol})^{0.9} * (\Lambda^{001})^{0.1} \qquad (19)$$
$$\Lambda_V^{HydLayer} = (\Lambda^{Ol})^{0.9} * (\Lambda^{010})^{0.1}$$

VIII. Dry olivine and intermediate serpentinization (30 $vol\%$ antigorite). Antigorite oriented with the [001] face horizontally, and the [010] face vertically

$$\Lambda_H^{HydLayer} = (\Lambda^{Ol})^{0.7} * (\Lambda^{001})^{0.3} \qquad (20)$$
$$\Lambda_V^{HydLayer} = (\Lambda^{Ol})^{0.7} * (\Lambda^{010})^{0.3}$$

IX. Dry olivine and high serpentinization (50 $vol\%$ antigorite). Antigorite oriented with the [001] face horizontally, and the [010] face vertically

$$\Lambda_H^{HydLayer} = (\Lambda^{Ol})^{0.5} * (\Lambda^{001})^{0.5} \quad (21)$$
$$\Lambda_V^{HydLayer} = (\Lambda^{Ol})^{0.5} * (\Lambda^{010})^{0.5}$$

X. Complete serpentinization (100 $vol\%$ antigorite). Antigorite oriented with the [001] face horizontally, and the [010] face vertically

$$\Lambda_H^{HydLayer} = \Lambda^{001} \quad (22)$$
$$\Lambda_V^{HydLayer} = \Lambda^{010}$$

Note S4. Density ρ

Pressure-dependent densities were calculated from equations of state (EoS) of relevant minerals reported in literatures. For each mineral, we took the cell volume data at high-P and we extrapolated a 4th order polynomial:

$$V_{cell}^{x}(P) = d_x P^4 + c_x P^3 + b_x P^2 + a_x P + V_{Ol}^{0} \quad (23)$$

The unit cell volume V_{Ol}^{cell}(Å^3) was then converted into molar volume V_{Ol}^{mol} $(cm^3\ mol^{-1})$ by employing the relation:

$$V_{mol}^{x}(P) = \frac{V_{cell}^{x}(P) * 6.022}{Z^x * 10} \quad (24)$$

Z^x indicates the fundamental cell units of a given mineral, and 6.022 is linked to Avogadro number ($N_A = 6.022 \times 10^{23}$). To compute T-dependent density we took the thermal expansion coefficient α of each mineral from literature.

$$\alpha^{Ol}(P,T) = \alpha_0^{Ol} + e_{Ol}T + f_{Ol}P + g_{Ol}PT + h_{Ol}P^2 \text{ (olivine)} \quad (25)$$
$$\alpha_{Atg} = \alpha_{Atg}^{0}$$

The reduction of cell volume caused by temperature increase ΔT is described by the relation:

$$V_{mol}^{x}(P,T) = \frac{V_{mol}^{x}(P)}{1 + \alpha^x(P,T) * \Delta T} \quad (26)$$

Density was finally obtained by using the molar weight $W_{mol}^{x}(g\ mol^{-1})$ of each mineral:

$$\rho^x(P,T) = \frac{W_{mol}^{x}}{V_{mol}^{x}(P,T)} * 1000 \quad (27)$$

Table S3 summarizes all coefficients. Data for olivine were taken from: $V_{cell}^{Ol}(P)$ (Downs et al., 1996), $\alpha^{Ol}(P,T)$ [20]. Antigorite data were taken from: $V_{cell}^{Atg}(P)$ [21], α^{Atg} [22].

We computed the density of each lithology as the weighted average of the constituting minerals (Figure S9d):

$$\rho_{AmbMan} = \rho_{LitSlab} = \rho_{Crust} = \rho^{Ol} \quad (28)$$
$$\rho_{Hyd} = (1 - \varphi_{Atg}) \cdot \rho_{Ol} + \varphi_{Atg} \cdot \rho_{Atg} \quad (29)$$

where φ_{Atg} indicates the volume fraction of antigorite in the rock.

Note S5. Specific Heat Capacity Cp

The specific heat capacity $Cp^x(J\,kg^{-1}K^{-1})$ of each mineral phase was obtained from the measurements of volumetric $Cp^x_{vol}(J\,cm^{-3}K^{-1})$ and molar $Cp^x_{mol}(J\,mol^{-1}K^{-1})$ heat capacities, see Table S3 for a summary of all coefficients.

We extrapolated olivine's specific heat capacity Cp^{Ol} from the dataset of Ref [20]:

$$Cp^{Ol}(P,T) = Cp^{Ol}_0 + a_{Ol}T^{-0.5} + b_{Ol}T^{-2} + c_{Ol}P + d_{Ol}P^2 \quad (30)$$

We extrapolated the specific heat capacity of antigorite Cp^{Atg} from the dataset of Ref [22]:

$$Cp^{Atg}(P,T) = f_{Atg}T^6 + e_{Atg}T^5 + d_{Atg}T^4 + c_{Atg}T^3 + b_{Atg}T^2 + a_{Atg}T^1 - Cp^{Atg}_0 \quad (31)$$

We computed the specific heat capacity of each lithology as the weighted average of the constituting minerals (Figure S9e):

$$Cp_{AmbMan} = Cp_{Crust} = Cp^{Ol} \quad (32)$$

$$Cp_{Hyd} = (1-\varphi_{Atg}) \cdot Cp_{Ol} + \textit{f}_{Atg} \cdot Cp_{Atg} \quad (33)$$

where φ_{Atg} indicates the volume fraction of antigorite in the rock.

Note S6. Code limitations:

Our model setting presents several limitations due the assumptions employed in the calculations:

(1) We computed the heat transfer equation for 2D case. The resulting slab can only be heated from four sides (left, right, bottom, top), and thus is expected to be colder than the 3D geometry (width is missing).

(2) We used a simplified petrology for the three lithologies of the slab, by assuming to be exclusively composed by olivine and antigorite. The aggregate thermal conductivity of each lithology should be calculated including the contribution of other major phases: clinopyroxenes and orthopyroxenes, garnets (basaltic crust and oceanic lithosphere); Al-rich phases (phlogopite, phengite, chlorite) and SiO_2-polymorphs (sediment blanket), other hydrous phases (e.g., talc, brucite, humite group). However, it should be considered that the lack of thermal conductivity datasets on major mantle minerals has been progressively filled by our recent measurements. It's only a matter of time before more comprehensive aggregate Λ estimates of mantle rocks will be available.

(3) The phase proportions in each lithology should vary accordingly to their *P*-*T* stability field. In our calculation we simply assumed that antigorite remains stable at any *P*-*T* condition. Future models should compute the breakdown of antigorite as the temperature increase.

(4) Our model is purely thermal, and subduction occurs with a constant sinking velocity. Future models should be thermo-mechanical, thus including buoyancy-driven subduction, and the variable sinking velocity should arise self-consistently from the interactions between the slab and the mantle.

(5) The contribution of additional heating source should be considered, e.g. radioactive elements (enriched in the basaltic crust and in the sediments), frictional heating, and positive/negative latent heat at the phase transitions.

(6) The domain in our model is limited to the slab, and a 500 m thick surrounding mantle. In nature, the slab heating causes the corresponding cooling of the mantle around the slab. Therefore, the temperature of the ambient mantle changes as the slab heats up. Future models should include larger volumes of the ambient mantle to reproduce this effect.

(7) In our model subduction starts at 120 km to avoid the interactions between the slab and the overriding plate. These interactions, however, influence the temperature field of the slab in the shallow portion of the subduction zone, thus influencing its *P*-*T*-t path before

being fully decoupled from the overriding plate. Future models should include these interactions.

(8) Corner-flow models[16] show that as the slab subducts there is an upwelling of hot material from deep mantle regions toward the mantle wedge. Moreover, part of the shallow cold mantle is dragged at depth by the subducting slab, thus creating a cold nose in the mantle wedge. This cold material can protect the slab from the high temperatures of the deep mantle, thus maintaining the slab surface cold for a longer time. Future models should also include the corner flow in the mantle wedge.

Note S7. Code benchmark:

We verified the reliability of the numerical model by comparing it against a known benchmark case. We designed a 2D model with Gaussian temperature distribution. Benchmark settings are summarized in Table S7. We solved the Fourier's heat diffusion law numerically and benchmark it against the analytical solution for the same problem setting (Fig. S10):

$$T(x,t) = \frac{T_{\max}}{\sqrt{1+\frac{4\kappa t}{\sigma^2}}} exp\left(\frac{-r^2}{\sigma^2+4\kappa t}\right) \tag{34}$$

$$r = \sqrt{x^2+y^2}$$

where T is the temperature as a function of position x and time t, $T_{\max}$ is the peak temperature in the Gaussian profile, κ is the thermal diffusivity ($\Lambda/(\rho * Cp)$), and σ the amplitude of the Gaussian distribution. To quantify the goodness of our discretization we computed the misfit between analytical and numerical solution, at a given node position. We further computed the $Misfit$ between the analytical T_a and numerical solution T_n, and the L^2 norm (Fig. S11):

$$Misfit = T_a - T_n \tag{35}$$

$$L^2 = \frac{\sqrt{\sum(Misfit * \Delta x * \Delta y)^2}}{X * Y}$$

where X is the total length of the domain and Δx the grid spacing. As reported in Fig. S10, the $L^2 << 1$, thus indicating a negligible misfit. The benchmarking proves the chosen discretization is sufficient to solve numerically the heat diffusion equation.

Note S8. Assumption of perfect alignment of single-crystalline antigorite along the slab dip angle:

Several field observations, deformation experiments, and theoretical calculations have reported that antigorite has a strong crystal preferred orientation (CPO), in which its [001] direction tends to orient perpendicularly to the shear direction. For example, Padrón-Navarta et al.[23] reported a strong foliation in natural serpentinites samples (Cerro del Almirez, Spain): antigorite's [001] shows a peak of relative density of 4.9–17.4 m.u.d. (multiples of uniform distribution) along the Z-axis, which is perpendicular to the shear direction (X-axis). Moreover, the majority of antigorite grains orient their [001] direction $\pm 30^{o}$ from the Z-axis. Similar behaviors have also been reported by Nishii et al.[24] (4.7–18 m.u.d.) and Jung[25] (5 m.u.d.). On these bases, it is reasonable to assume that, during subduction, antigorite would orient its [001] direction normal to the main shear direction, i.e., along the slab dip.

Unfortunately, it is not possible to find ophiolites of subducted slab which maintained the CPO gained during subduction. Moreover, to our knowledge, it is not yet possible to infer the orientation of antigorite inside the slab from seismology. Satta et al.[26] reported that in order to see 1 second delay on the shear-velocity, the seismic wave has to cross a >10 km-long path of fully serpentinized rocks (100% alteration), and the incident angle of the seismic wave must be parallel to the foliation plane. Given such conditions and the limited time sensitivity of modern seismometers (~ 10 milliseconds), it is almost impossible to tell the orientation of a partially serpentinized rock layer (<70% alteration) (see Allen et al.[27] and Cooper et al.[28]) which is crossed by a non-ideally oriented seismic wave for less than 10 km. Therefore, it is reasonable to assume that antigorite's [001] direction is oriented perpendicularly to the slab dip within $\pm 30^{o}$.

Moreover, our TDTR measurements report the upper and lower bounds of antigorite's thermal conductivity depending on the orientation of the crystal. We can define φ as the incident angle between the heat flux and the foliation plane of antigorite. On one hand, if the [001] direction is parallel to the heat flux (i.e., perpendicular to the {001} foliation planes φ = 90°), the heat will flow slowly because of the low Λ^{001}. On the other hand, if the [001] direction is perpendicular to the heat flux (i.e., parallel to the {001} foliation planes $\varphi = 0^{o}$), the heat will flow faster because of the high Λ^{010}. If the antigorite is oriented with an incident angle between $0^{o} < \varphi < 180^{o}$ and $\varphi \neq 90^{o}$, we can assume that the thermal conductivity would be a value between Λ^{001} and Λ^{010}. In this case, we can use a simple function:

$$\Lambda_{lat}^{atg}(\varphi) = (\Lambda^{001})^{sin\varphi} * (\Lambda^{010})^{1-sin\varphi} \quad (36)$$

Here we used a geometrical average because Λ^{001} is much lower than Λ^{010}, and hence it will act as a bottleneck to the lattice heat transport. With this equation we can calculate antigorite's thermal conductivity as a function of its orientation with respect to the heat flux (Table S8, Fig. S13).

Equation 36 is a fair approximation of the thermal conductivity of antigorite when 0º $\leq \varphi \leq$ 180º: Λ_{lat}^{atg} remains low for high incidence angle (45º $\leq \varphi \leq$ 135º) because heat would flow through several foliation planes. However, Λ_{lat}^{atg} is high when the incidence angle is low ($\varphi \leq$ 30º or $\varphi \geq$ 150º) because heat would flow on the foliation planes, see Fig. S13.

Heat flux is always oriented along the direction of the maximum temperature gradient, which, in our case, is perpendicular to the slab surface (Fig. S6). Therefore, we can assume that the incident angle between heat flux and antigorite's foliation planes is high: φ = 90º$\pm$30º. This range of orientations does not show a major difference in antigorite's thermal conductivity: ~20% at ambient pressure which becomes less than 10% at high pressure (see the plateau of Λ^{001} in Fig. 1 of the main text). Even at φ = 30º and φ = 120º, the $\Lambda^{010}/\Lambda^{001}$ anisotropy remain large: ~3.4 at ambient pressure, and ~2.3 at *P*=7 GPa. For these reasons, we believe that the assumptions made to design our simplified model of heat flow through an anisotropic medium are sufficient to describe the physics of the phenomenon, and our conclusions are still valid even if antigorite is not perfectly aligned.

Note S9. Potential effects of grain boundary:

As we show in the main text, our single-crystal data along the *b*- and *c*-axis provide important basis that brackets the thermal conductivity of polycrystals. Its applicability to estimate the “average” thermal conductivity of a rock that likely contains polycrystals, depends on many factors, such as the realistic fraction of crystals along *b*- and *c*-axis. This, in turn, is influenced by: 1) the grain size; 2) the misorientation of the polycrystalline aggregate with respect to the local stress state; 3) the presence of other phases in the aggregate; 4) the large-scale stress field imposed by the tectonic settings.

As we discuss in Note S8, it has been reported that the maximum orientation density of the [001] direction along the Z-axis is ~4.7–18 m.u.d., and most of the grains orient their [001] within a ±30º angle with respect to the Z-axis. The thermal conductivity difference between a fully aligned aggregate and a slightly misaligned one is ~8–20%, whereas its anisotropy remains high (~2.3–3.4). In order to compute the aggregate thermal conductivity of an antigorite mineral assemblage, one has to determine: 1) the distribution density of the [001] orientation with respect to the Z-axis (which should be less than the ~4.7–18 m.u.d peak); and 2) the grain size of each crystal. With this information it is then possible to compute a weighted average considering what percentage of the bulk volume is oriented in which direction. There are codes (e.g., D-Rex[29]), which allow to compute the orientation and the grain size of crystals produced by an external stress field. These codes, however, are limited to olivine and enstatite slip systems. Implementing antigorite slip systems in these codes to produce CPO pole figures is beyond the scope of this paper. Our simplified approach is sufficient to prove the importance of antigorite anisotropy on slab’s thermal evolution.

The presence of misaligned grain boundaries can generate additional scattering of the heat-carrying phonons and result in a thermal resistance, i.e., thermal conductivity reduction[30]. The scattering due to tilt boundaries, however, seems to be minor with respect to scattering in the inter-grain regions, which is where the crystallographic disorder concentrates[30]. Moreover, it has been shown that the thermal resistance across the basal plane of mica (a phyllosilicate like antigorite) decreases as the planes are forced together by the increasing pressure[31]. As suggested by Wood[32], the fall in mica thermal conductivity reported by Powell and Griffiths[31] is attributed to the grain size reduction due to the crushing of the sample at high pressure. From these experimental evidences and theoretical considerations, it seems that phonon scattering at the grain boundaries of a phyllosilicates aggregate is mostly affected by the grain size rather than the misalignment of the crystals.

We therefore expect that the misalignment of antigorite crystals in a serpentinized rock has a minor impact on the aggregate thermal conductivity of the mineral assemblage, because 1) the misalignment is within a narrow angle ($\pm30^{o}$) and 2) the misalignment tends to reduce at high pressure and under high shear stress.

It will be interesting to investigate the effects of grain size on antigorite thermal conductivity. Padrón-Navarta et al.[23] report a natural sample of serpentinite with a grain size of few tens of μm, which is at the same order of magnitude of the single crystal samples we measured. If the reduction of thermal conductivity due to smaller grain size is proved experimentally in the future, it would further strengthen our point: a fine-grain aggregate of aligned antigorite crystals produced by an external shear stress field would create an even better thermally insulating layer. Thus, the antigorite's anisotropic thermal conductivity is expected to play an important role in influencing the temperature evolution of the slab down to ~250 km depth.

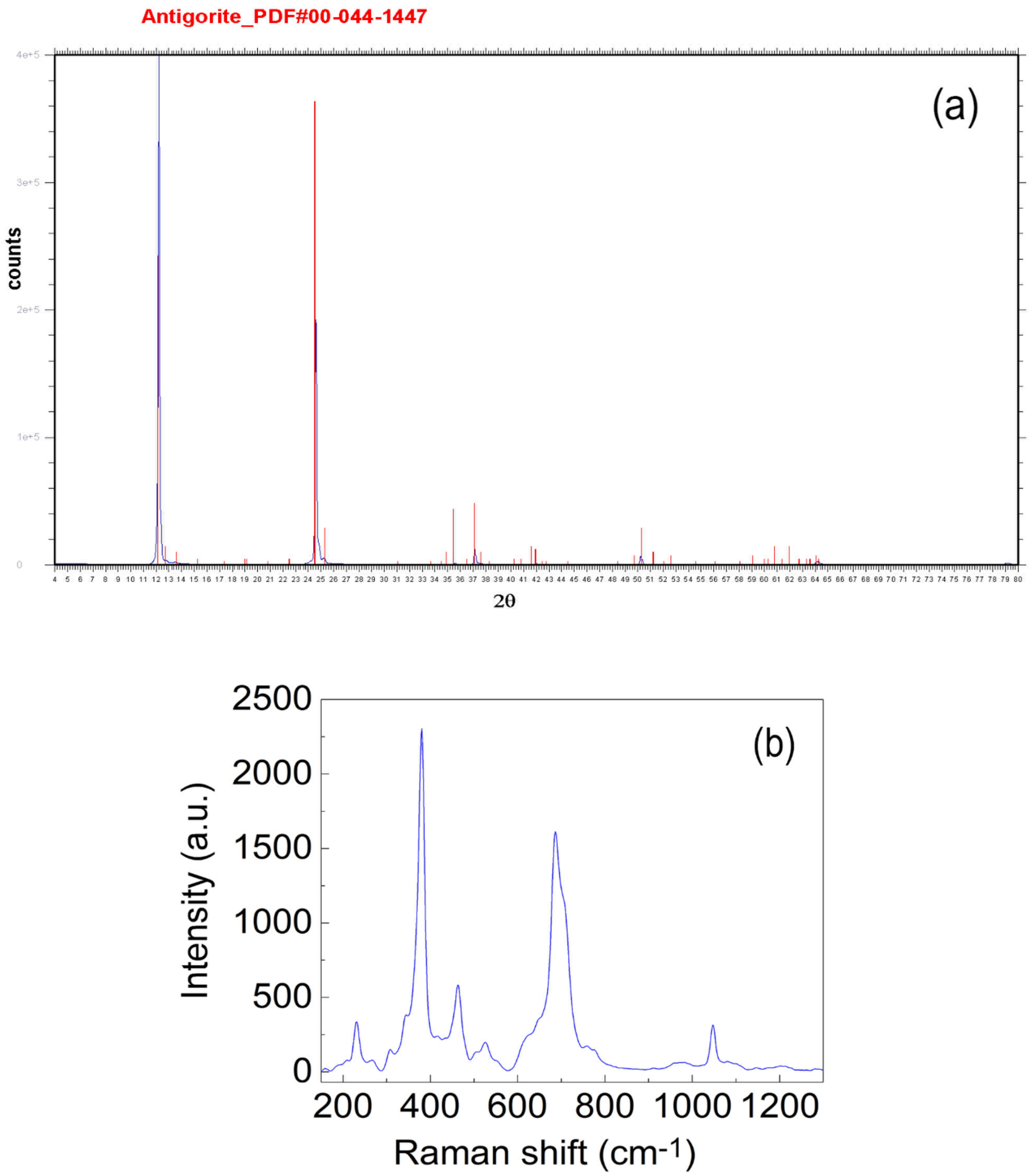

Figure S1. Representative (a) X-ray diffraction and (b) Raman spectrum of our natural antigorite sample. In (a), the blue curve is our measurement result and the red peaks are from antigorite's X-ray diffraction PDF database. In (b), the Raman intensity is in arbitrary unit (a.u.).

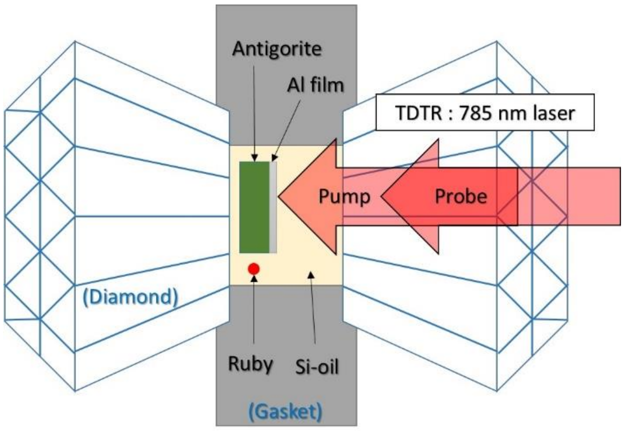

Figure S2. Schematic drawing of the high-pressure TDTR experimental setup in a DAC at room temperature. A single-crystal antigorite coated with Al film was placed in the DAC chamber. A ruby ball and silicone oil were used as the pressure calibrant and the pressure transmitting medium, respectively. The thermal conductivity of antigorite was measured by optical pump-probe method (see the main text for the details).

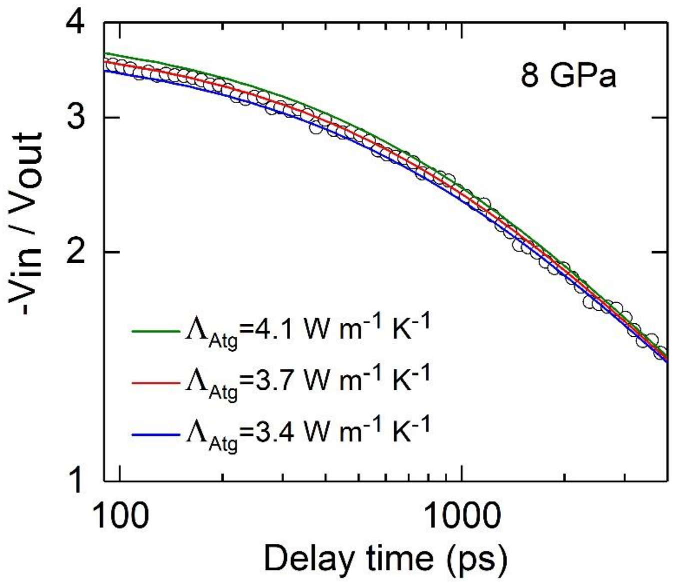

Figure S3. Example TDTR spectrum (open circles) for antigorite at 8 GPa and room temperature along *c*-axis loaded with Ar as the pressure medium. The data are fitted by thermal model calculations (color solid curves). Using the input parameters listed in Table S6, Λ_{Atg}=3.7 W m^{-1} K^{-1} (red curve) offers a best-fit to the raw spectrum. The ratio $-V_{in}/V_{out}$ is most sensitive to antigorite's thermal conductivity at few hundred picosecond (ps) delay time [33,34]. A 10% test error for Λ_{Atg} (green and blue curves) causes the fitting curve deviating from the data. Such high sensitivity demonstrates that based on the high-quality data, our thermal model fitting and the obtained Λ_{Atg} are precise and reliable.

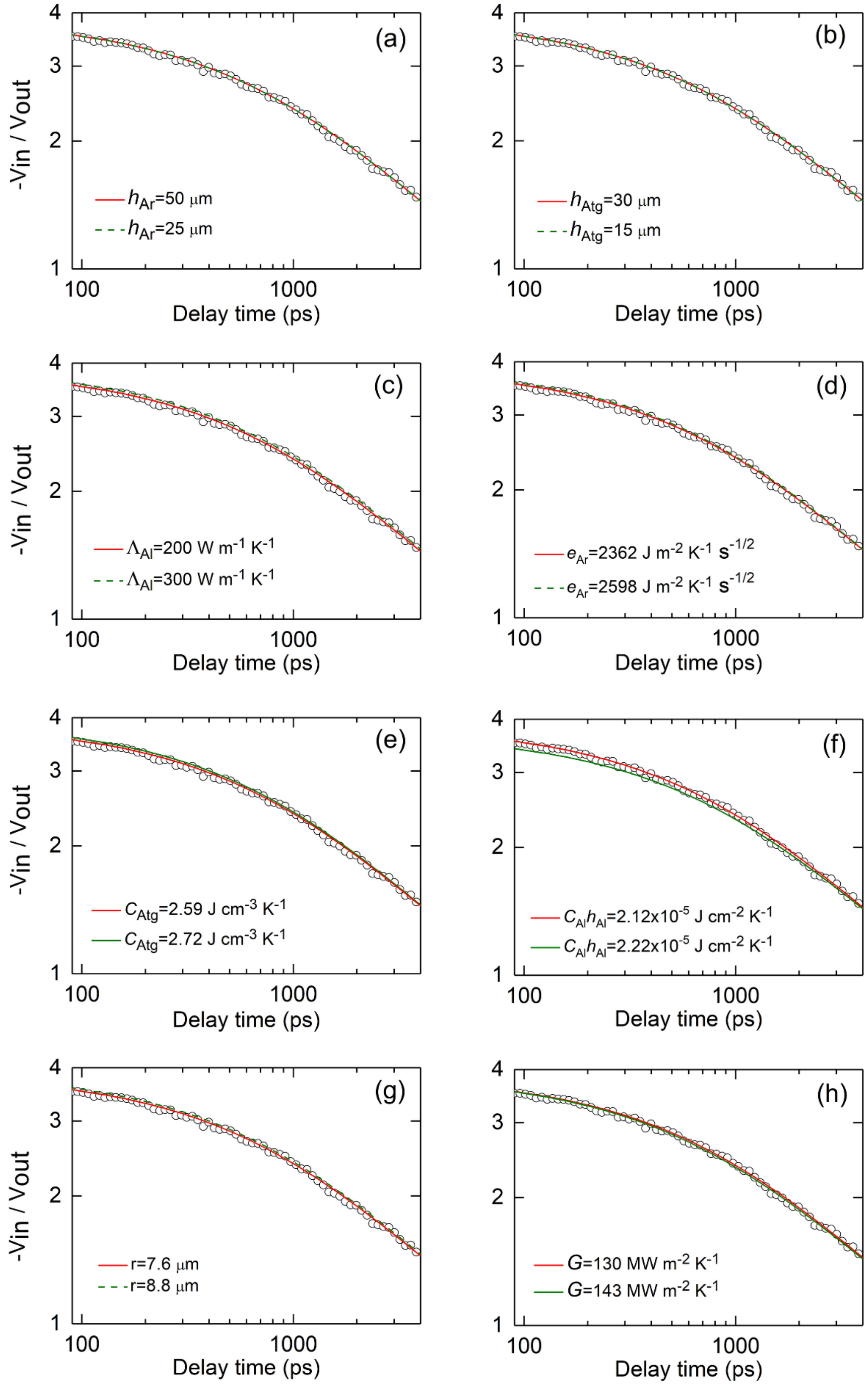

Figure S4. Sensitivity tests of the thermal model calculations to input parameters for antigorite at 8 GPa and room temperature loaded with Ar as the pressure medium. Here antigorite's thermal conductivity, Λ_{Atg}, is fixed at 3.7 W m^{-1} K^{-1}, as derived from Fig. S3, using parameters listed in Table S6. (**a**) and (**b**) Even if the thicknesses of Ar (h_{Ar}) and antigorite (h_{Atg}) change

by as much as 50%, the model calculations remain the same, i.e., uncertainties in their thicknesses do not affect our derived Λ_{Atg}. (**c**) Even if the high thermal conductivity of Al increases with pressure by 50%, it has very minor effect on the Λ_{Atg}. (**d**) Assuming an example uncertainty of 10% for the thermal effusivity of the pressure medium Ar, $e=(\Lambda_{Ar}C_{Ar})^{1/2}$, the Λ_{Atg} needs to decrease to 3.6 W m^{-1} K^{-1} to re-fit the data, i.e., propagating ~3% uncertainty. (**e**) Assuming the volumetric heat capacity of antigorite, C_{Atg}, has an error of ~5%, a Λ_{Atg}=3.6 W m^{-1} K^{-1} is required to re-fit the data, i.e., translating ~3% error. (**f**) The major uncertainty is from the uncertainty in the volumetric heat capacity of Al film per unit area—product of volumetric heat capacity and thickness, C_{Al} h_{Al}. Under our experimental condition of modulating the pump beam at 8.7 MHz, the ratio $-V_{in}$ /V_{out} at few hundred picosecond delay time scales inversely with the C_{Al} h_{Al}, see [33] for details. Assuming an example 5% uncertainty for C_{Al} h_{Al}, to fit the data the Λ_{Atg} needs to increase to 4.1 W m^{-1} K^{-1}, i.e., ~10% uncertainty. (**g**) If the laser spot size is uncertain by 15%, the model calculation is hardly affected, i.e., propagating minor error to the Λ_{Atg}. (**h**) If the thermal conductance of Al/antigorite and Al/Ar interfaces, G, is off by 10%, it has essentially no change in the model calculation.

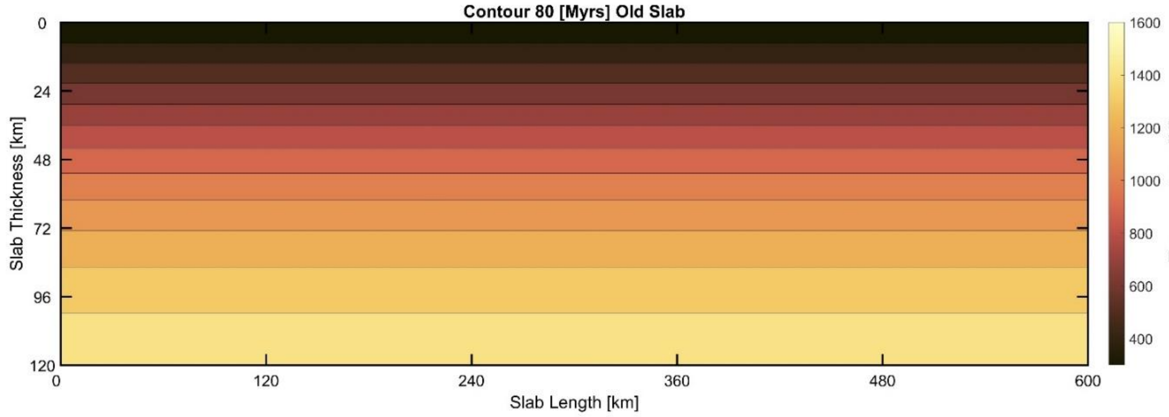

Figure S5. Schematics of the slab used in our models. The slab was simplified as a $120 \times 600\ km$ rectangle. The colour map represents the initial temperature of the slab: dim colours represent areas with low T, whereas bright colours indicate high T. To plot temperature, we used the Scientific Colour Maps '*lajolla*'.

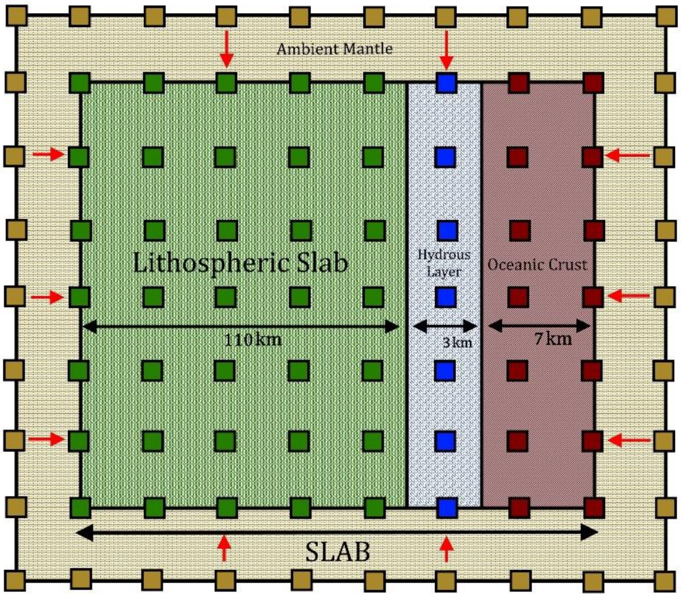

Figure S6. Schematics of slab lithologies. We subdivided the slab into different domains: oceanic crust (7-km-thick), hydrous layer (3-km-thick), and lithospheric slab (110-km-thick). Each lithology is characterized by a different mineral assemblage (see main text). We added an extra stencil of nodes at the four sides of the slab to represent the ambient environment (surface lithosphere or deep mantle). The red arrows indicate the heat flow from the hot ambient mantle toward the cold slab.

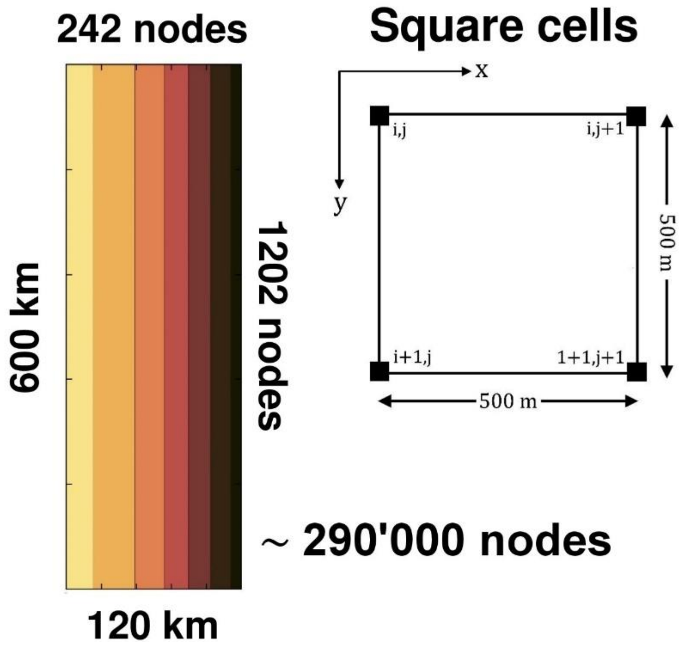

Figure S7. Schematics of discretization of the slab. We simplified the slab as a $120 \times 600\ km$ rectangle, and we discretized the domain using square grid cells. Each cell is bounded by 4 nodes at the corners with a constant grid spacing of $500\ m$. We used the $[i, j]$ coordinate system to identify each node: i identifies the rows (y-direction) and j identifies the columns (x-direction). We used a Scientific Colour Map to plot the temperature inside the slab (see caption of Fig. S5).

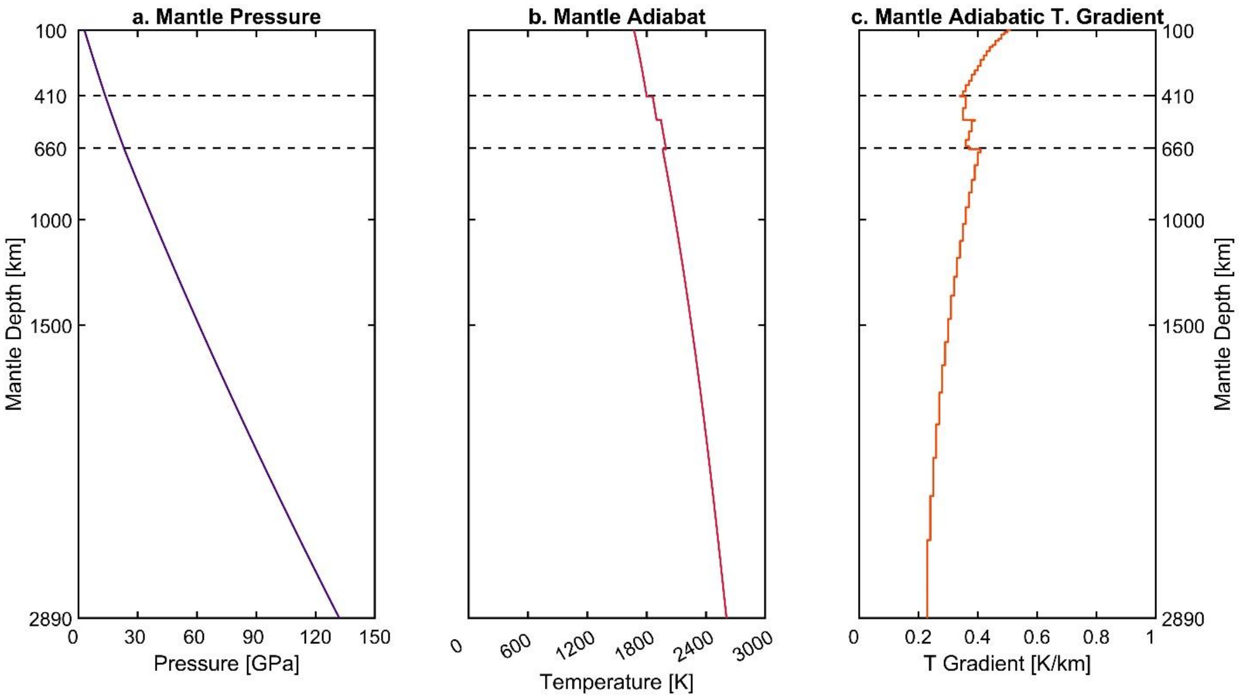

Figure S8. (a) Pressure profile of the mantle[7]. (b) Adiabatic temperature profile in the mantle[8]. (c) Temperature gradient in the mantle[8].

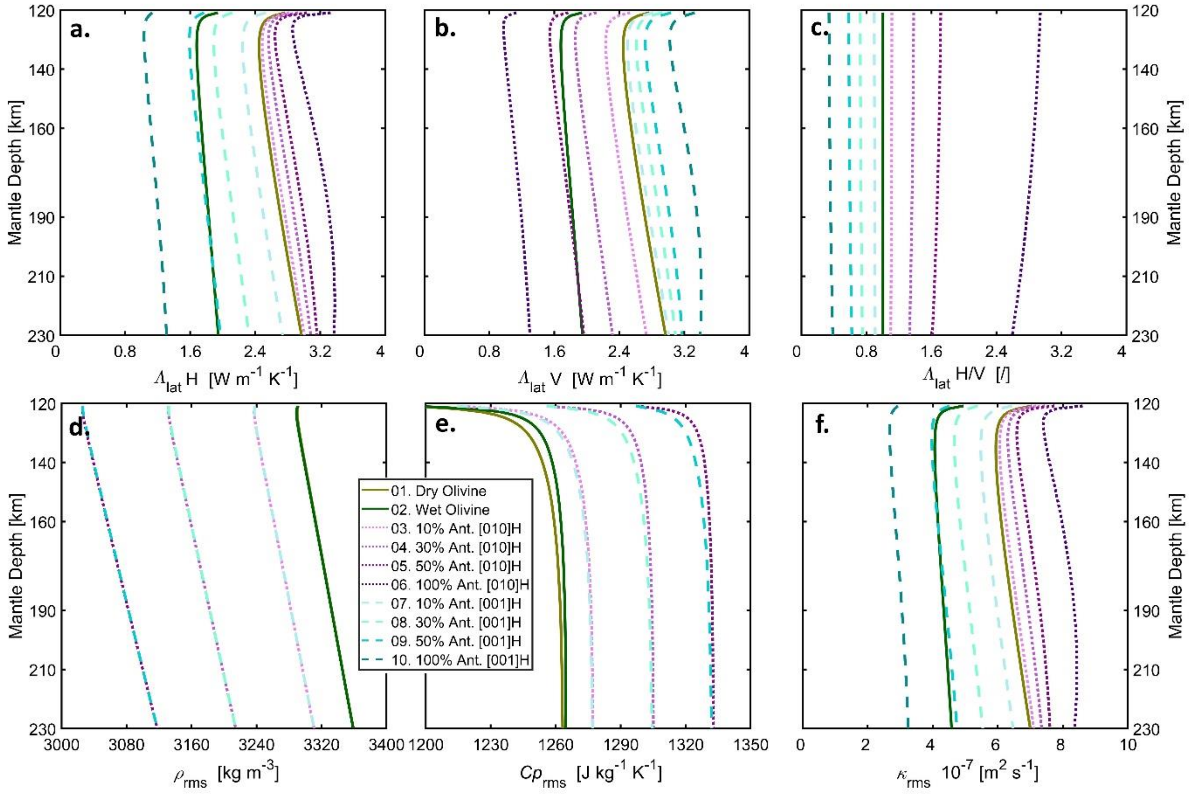

Figure S9. (a) Horizontal lattice thermal conductivity. (b) Vertical lattice thermal conductivity. (c) Thermal conductivity ratio (horizontal/vertical). (d) Density. (e) Specific heat capacity. (f) Thermal diffusivity.

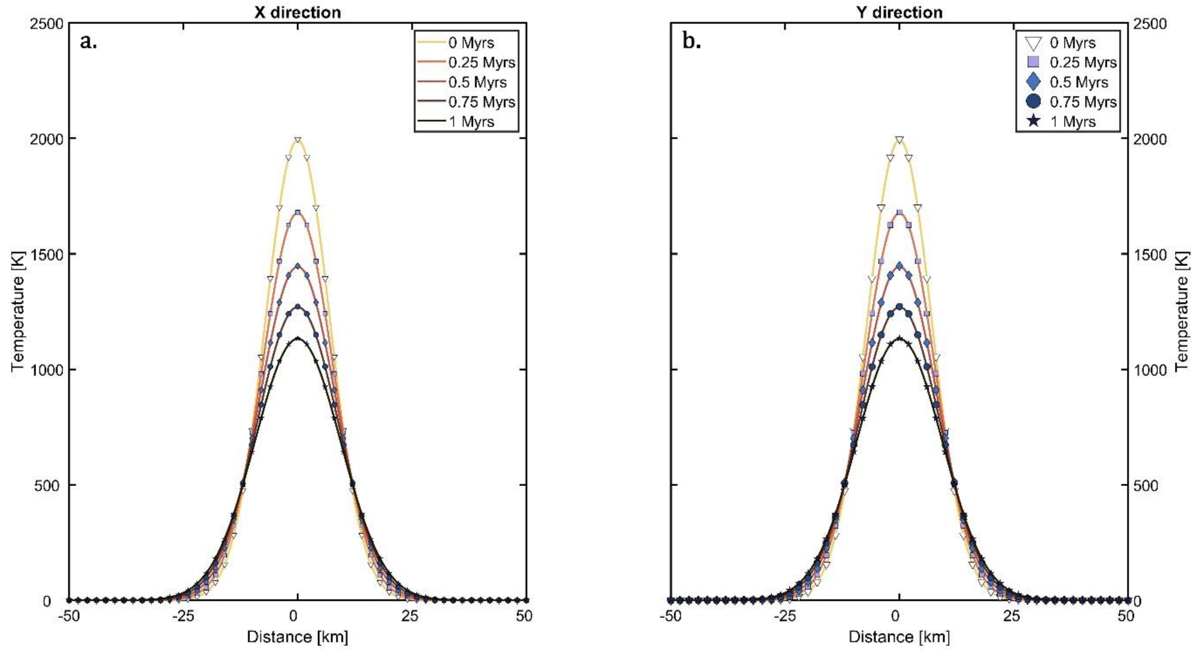

Figure S10. Benchmark: comparison between analytical and numerical solutions. The two subplots illustrate two perpendicular transect of the 2D Gaussian distribution of temperature: (a) along x-direction; (b) along y-direction. In the figure are shown 5 different snapshots of the temperature evolution (0;0.25;0.5;0.5;1 Myrs). We used a Scientific Colour Map (see caption of Fig. S5) to plot the progressive cooling of the domain from bright (hot) to dim (cold). The solid lines represent of the analytical solutions, whereas the e markers indicate the value of the numerical solution at the corresponding node (every 50th nodal point is plotted here).

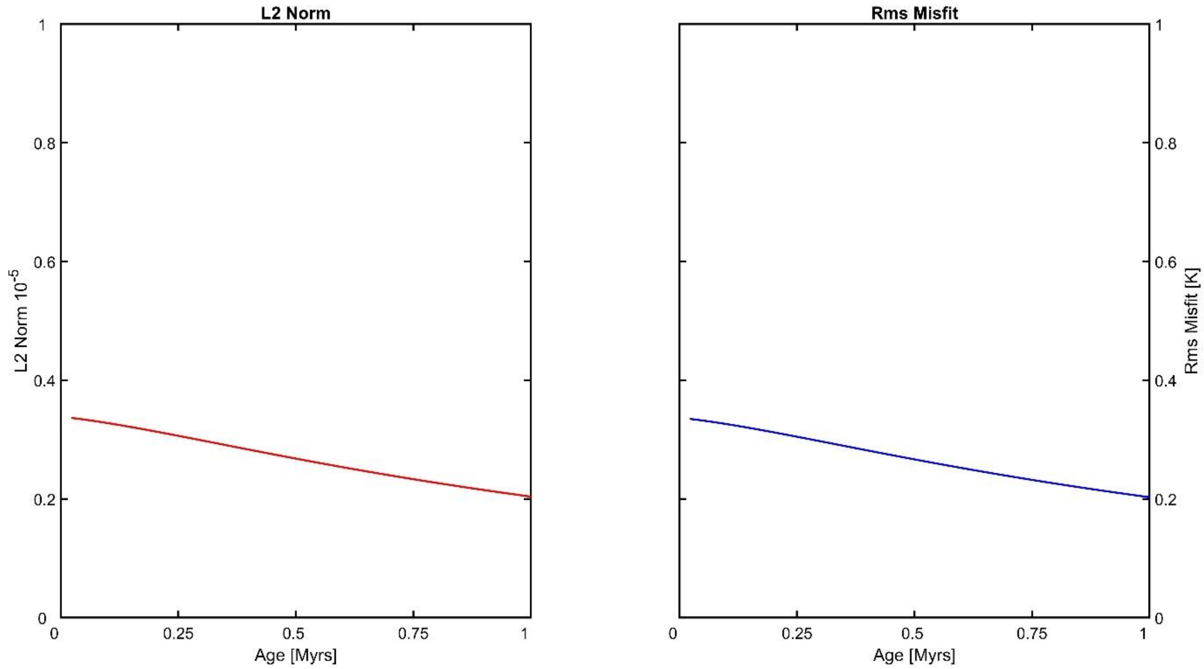

Figure S11. Benchmark: (a) L^2 norm evolution over 1 Myrs of simulated time, (b) misfit between the analytical and numerical solution.

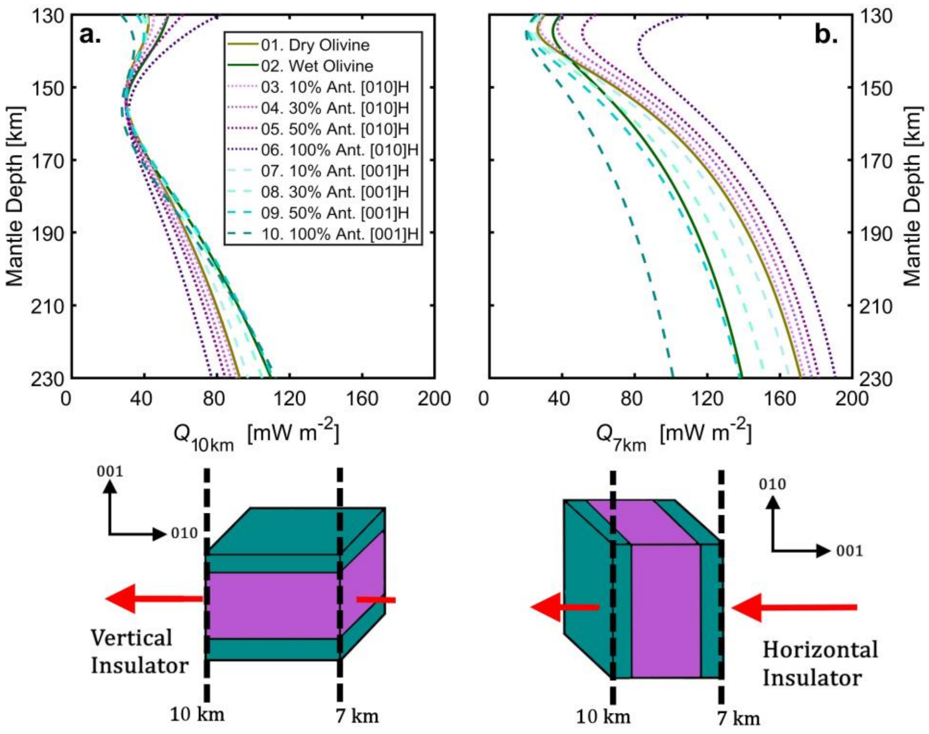

Figure S12. Radial profile of heat flux Q ($mW\ m^{-2}$) computed at both sides of hydrous layer. (a) Internal side (10 km from slab surface). (b) external side (7 km from slab surface). In each model, we used the Fourier Law of heat conduction to compute the heat flux: $Q = -\Lambda \nabla T$. Each line indicates the Q profile of one model: green solid lines for set 1 as references, dotted purple lines for set 2 with vertical insulator, and dashed teal lines for set 3 with horizontal insulator. The two boxes below represent the two configurations of antigorite used in the model (see Fig.3 in the main manuscript), while the vertical black dashed lines indicate the two sides of the hydrous layer. The heat flux entering the hydrous layer from the external side (Q_{7km}, Fig. 5b) is higher in the model set 2 (vertical insulator, purple dotted lines) compared to model set 3 (horizontal insulator, teal dashed lines), $Q_{7km}^{010} > Q_{7km}^{001}$. In the Λ_H^{001} configuration, $Q_{10km}^{010} > Q_{10km}^{001}$ (Fig. 5a) at shallow depth due to the low thermal conductivity, but it becomes $Q_{10km}^{010} < Q_{10km}^{001}$ at greater depth because the hydrous layer is more thermally insulated than the Λ_H^{010} configuration. Compared to antigorite, the heat flux through the hydrous layer in the dry olivine reference (Q_{7km}^{DryOl}, solid olive-green line in Fig. 5b) is higher than Q_{7km}^{001} (horizontal insulator), but lower than Q_{7km}^{010} (vertical insulator), because the thermal conductivity of antigorite along both direction is $\Lambda^{010} > \Lambda^{DryOl} > \Lambda^{001}$.

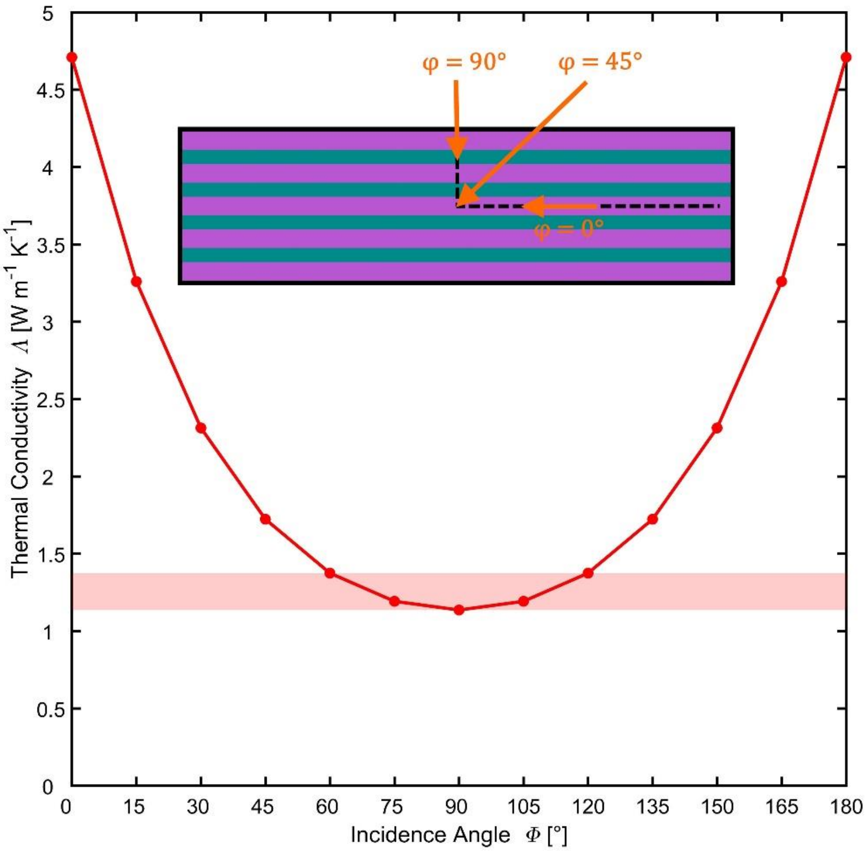

Figure S13. Antigorite lattice thermal condutivity Λ_{lat}^{atg} $(W\ m^{-1}\ K^{-1})$ as a function of the incidence angle φ between the heat flux and antigorite's foliation planes {001}. The thermal conductivity of each marker (red dots) was computed using eq. 36: $\Lambda_{lat}^{atg}(\varphi) = (\Lambda^{001})^{sin\varphi} * (\Lambda^{010})^{1-sin\varphi}$. This empirical equation can be use to compute Λ_{lat}^{atg} of non ideally oriented antigorites crystals (see Table S8). We used our TDTR measurements to set the two endmembers values of antigorite's thermal conductivity: cross-plane Λ^{001} (min); and in-plane Λ^{010} (max). The trend reproduced by eq. 36 (red line) can be physically justified by the following geometric considerations: 1) Λ_{Atg} is minimum at $\varphi = 90°$, because the heat flows through all foliation planes, i.e. parallel to the [001] direction $\Lambda_{lat}^{atg} = \Lambda^{001}$. Note that, for high incidence angles ($60° < \varphi < 120°$), a small misalignment of $\varphi = \pm 30°$ results in $\Lambda_{lat}^{atg} \sim \Lambda^{001} + 0.25\ W\ m^{-1}\ K^{-1}$ (red area). 2) Λ_{lat}^{atg} is maximum at $\varphi = 0°$ and $\varphi = 180°$, because the heat flows on the foliation plane, i.e. parallel to the [010] direction $\Lambda_{lat}^{atg} = \Lambda^{010}$. In this case, at low incidence angles ($0° < \varphi < 30°$ and $150° < \varphi < 180°$), a small misalignment of $\varphi = \pm 30°$ results in $\Lambda_{lat}^{atg} \sim \Lambda^{010} - 2\ W\ m^{-1}\ K^{-1}$. In this figure are reported only the values at ambient P,T. The layered structure inside the plot represents the heat flowing through an antigorite crystal: the orange arrows indicates the heat flow with different incidence angles ($\varphi = 90°, 45°, 0°$); the purple areas represent antigorite planes characterised by high thermal conductivity Λ^{010}; the teal areas represent the cross-plane regions characterized by low thermal conductivity Λ^{001}.

Depth Range	a	b	c
$\mathbf{0-15\ km}$	-4.6499×10^{-2}	2.5506×10^{-2}	0
$\mathbf{15-24.4\ km}$	-9.0644×10^{-2}	2.8449×10^{-2}	0
$\mathbf{24.4-80\ km}$	-2.0601×10^{-1}	3.3189×10^{-2}	-4.7197×10^{-7}
$\mathbf{80-220\ km}$	-2.0626×10^{-1}	3.3197×10^{-2}	-5.3320×10^{-7}
$\mathbf{220-400\ km}$	-1.9571×10^{-1}	3.2387×10^{-2}	2.9291×10^{-6}
$\mathbf{400-600\ km}$	-3.7043×10^{-1}	3.1522×10^{-2}	6.1821×10^{-6}
$\mathbf{600-670\ km}$	-2.2151	3.7621×10^{-2}	1.1420×10^{-6}
$\mathbf{670-771\ km}$	-3.8230	3.8728×10^{-2}	3.0723×10^{-6}
$\mathbf{771-2741\ km}$	-4.3415	3.9731×10^{-2}	2.5861×10^{-6}
$\mathbf{2741-2891\ km}$	-5.1590	4.0458×10^{-2}	2.4261×10^{-6}

Table S1. Coefficients to compute the mantle pressure profile.

Thermal Conductivity Coefficients						
	a_x	b_x	c_x	d_x	Λ_x^0	z_x
$\mathbf{Ol_{Dry}}$	$-$	$-$	-8.0102×10^{-3}	4.0779×10^{-1}	3.6000	0.5
$\mathbf{Ol_{Wet}}$	-2.2204×10^{-16}	1.0658×10^{-14}	-8.1600×10^{-3}	3.5683×10^{-1}	2.4531	0.5
$\mathbf{Atg_{010}}$	2.3825×10^{-3}	-6.6405×10^{-2}	5.1957×10^{-1}	-7.2042×10^{-1}	4.7093	0.5
$\mathbf{Atg_{001}}$	2.6070×10^{-4}	-8.6826×10^{-3}	7.1635×10^{-2}	1.0617×10^{-1}	1.1366	0.5

Table S2. Coefficients for the thermal conductivity parameterization. Abbreviations stands for: dry olivine (Ol_{Dry}), wet olivine (Ol_{Wet}), quartz (Qtz), antigorite [010] (Atg_{010}), antigorite [001] (Atg_{001}).

Unit Cell Volume								
	a_x	b_x	c_x	d_x	V_0^x [Å^3]	Z_n	$W_x^{mol}[g\ mol^{-1}]$	Reference
**Ol**	-3.4187	3.3457×10^{-1}	-2.4866×10^{-2}	6.3936×10^{-4}	290.18	4	140.69	(Downs et al., 1996)
**Atg**	-53.966	7.8034	-1.0820	5.2566×10^{-2}	2927.4	17	274.68	[21]
**Thermal expansion coefficient**								
	e_x	f_x	g_x	h_x	$\alpha_0^x\ [K^{-1}]$	$-$	$-$	Reference
**Ol**	1.695×10^{-8}	-1.030×10^{-6}	-7.907×10^{-10}	2.286×10^{-8}	2.627×10^{-5}	$-$	$-$	[20]
**Atg**	$-$	$-$	$-$	$-$	2.65×10^{-5}	$-$	$-$	[22]
**Heat Capacity**								
	a_x	b_x	c_x	d_x	e_x	f_x	Cp_0^x	Reference
**Ol**	-1.238×10^{4}	-3.139×10^{6}	-3.184	8.414×10^{-2}	$-$	$-$	$1585\ J\ kg^{-1}K^{-1}$	[20]
**Atg**	6.6654	-1.0486×10^{-2}	8.3786×10^{-6}	-3.6011×10^{-9}	7.9288×10^{-13}	-7.0168×10^{-19}	$276.85\ J\ kg^{-1}K^{-1}$	[22]

Table S3. Coefficients to compute unit cell volume, thermal expansion coefficients, and heat capacity.

Model	Λ_H	Λ_V	φ_{Atg}
Reference			
1	Λ^{DryOl}	Λ^{DryOl}	0.0
2	Λ^{WetOl}	Λ^{WetOl}	0.0
Set 1			

3	Λ^{Atg010}	Λ^{Atg001}	0.1
4	Λ^{Atg010}	Λ^{Atg001}	0.3
5	Λ^{Atg010}	Λ^{Atg001}	0.5
6	Λ^{Atg010}	Λ^{Atg001}	1.0
Set 2			
7	Λ^{Atg001}	Λ^{Atg010}	0.1
8	Λ^{Atg001}	Λ^{Atg010}	0.3
9	Λ^{Atg001}	Λ^{Atg010}	0.5
10	Λ^{Atg001}	Λ^{Atg010}	1.0

Table S4. Antigorite configuration

Model	$D_{700}[km]$ $7\ km$	$D_{700}[km]$ $9\ km$	$D_{700}[km]$ $11\ km$	$D_{1000}[km]$ $7\ km$	$D_{1000}[km]$ $9\ km$	$D_{1000}[km]$ $11\ km$
1	162.4	179.4	194.4	213.4	217.9	219.9
2	156.4	174.4	199.9	205.9	217.4	220.9
3	163.4	179.9	194.9	213.9	217.9	219.9
4	164.4	180.9	194.9	214.4	218.4	219.9
5	165.4	181.9	195.4	215.4	218.9	220.4
6	168.9	184.4	195.9	216.4	219.4	220.9
7	160.9	178.4	195.4	212.4	217.9	219.9
8	158.4	176.4	197.4	209.4	217.4	220.4
9	155.9	174.4	200.4	204.9	216.9	220.4
10	152.4	171.4	206.9	190.4	215.9	221.4

Table S5. Maximum depth of the 700 K and 1000 K isotherms.

P (GPa)	C_{Atg} (J cm^{-3} K^{-1})	C_{Al} (J cm^{-3} K^{-1})	h_{Al} (nm)	$e=(\Lambda_{Ar}C_{Ar})^{1/2}$ (J m^{-2} K^{-1} s$^{-1/2}$)	r (µm)	$h_{Atg/Ar}$ (µm)	Λ_{Al} (W m^{-1} K^{-1})	G (MW m^{-2} K^{-1})
8	2.59	2.54	83.8	2362	7.6	30/50	200	130

Table S6. Input parameters in the thermal model for antigorite at 8 GPa and 300 K along *c*-axis in TDTR measurements. In this experimental run, the Al thickness at ambient pressure is 86 nm. C_{Atg}: antigorite heat capacity, C_{Al}: Al heat capacity, h_{Al}: Al thickness, e: Ar thermal effusivity, r: laser spot size, h_{Atg}: antigorite thickness, h_{Ar}: Ar thickness, Λ_{Al}: Al thermal conductivity, G: thermal conductance of Al/antigorite and Al/Ar interfaces.

**Parameter**	**Symbol**	**Value**
**Peak Temperature**	T_{max}	$2000\ K$
**Gaussian Amplitude**	σ	$10000\ m$
**Density**	ρ	$3300\ kg\ m^{-3}$
**Specific Heat Capacity**	Cp	$1200\ J\ kg^{-1}\ K^{-1}$
**Thermal Conductivity**	Λ	$3\ W\ m^{-1}\ K^{-1}$
**Horizontal Length**	X	100 km

**Vertical Length**	Y	100 km
**Grid Nodes**	n	201x201
**Grid Spacing**	Δx	$500\,m$
**Grid Spacing**	Δy	$500\,m$
**Time Step**	Δt	$2000\,yrs$

Table S7. Parameters used to compute the analytical solution for a Gaussian distribution of the temperature field

φ	90 º	80 º	60 º	45 º	30 º	10 º	0 º
$\Lambda_{lat}^{atg}\ [W\ m^{-1}\ K^{-1}]$	1.14	1.16	1.38	1.72	2.31	3.68	4.71
φ	90 º	100 º	120 º	135 º	150 º	170 º	180 º
$\Lambda_{lat}^{atg}[W\ m^{-1}\ K^{-1}]$	1.14	1.16	1.38	1.72	2.31	3.68	4.71

Table S8. Antigorite thermal conductivity Λ_{lat}^{atg}, computed for different incidence angles φ between the heat flow and the foliation planes {001}. Each thermal conductivity was computed using eq. 36: $\Lambda_{lat}^{atg}(\varphi) = (\Lambda^{001})^{sin\varphi} * (\Lambda^{010})^{1-sin\varphi}$. This empirical equation can be use to compute Λ_{lat}^{atg} of non ideally oriented antigorites crystals.

Supplementary References

1. Turcotte, D. L. & Schubert, G. *Geodynamics*. (Cambridge University Press, 2014). doi:doi.org/10.1017/CBO9780511843877.
2. Stein, C. A. & Stein, S. A model for the global variation in oceanic depth and heat flow with lithospheric age. *Nature* **359**, 123–129 (1992).
3. Grevemeyer, I., Ranero, C. R. & Ivandic, M. Structure of oceanic crust and serpentinization at subduction trenches. *Geosphere* **14**, 395–418 (2018).
4. John, T., Scambelluri, M., Frische, M., Barnes, J. D. & Bach, W. Dehydration of subducting serpentinite: Implications for halogen mobility in subduction zones and the deep halogen cycle. *Earth Planet. Sci. Lett.* **308**, 65–76 (2011).
5. Irifune, T. & Ringwood, A. E. Phase transformations in a harzburgite composition to 26 GPa : implications for dynamical behaviour of the subducting slab. *Earth Planet. Sci. Lett.* **86**, 365–376 (1987).
6. Wei, S. S., Wiens, D. A., van Keken, P. E. & Cai, C. Slab temperature controls on the Tonga double seismic zone and slab mantle dehydration. *Sci. Adv.* **3**, e1601755 (2017).
7. Dziewonski, A. M. & Anderson, D. L. Preliminary reference Earth model. *Phys. Earth Planet. Inter.* **25**, 297–356 (1981).
8. Katsura, T. A Revised Adiabatic Temperature Profile for the Mantle. *J. Geophys. Res. Solid Earth* **127**, e2021JB023562 (2022).
9. Gerya, T. *Introduction to numerical geodynamic modelling*. (Cambridge University Press, 2019).
10. Xu, Y. *et al.* Thermal diffusivity and conductivity of olivine , wadsleyite and ringwoodite to 20 GPa and 1373 K. *Phys. Earth Planet. Inter.* **144**, 321–336 (2004).
11. Zhang, Y., Yoshino, T., Yoneda, A. & Osako, M. Effect of iron content on thermal

conductivity of olivine with implications for cooling history of rocky planets. *Earth Planet. Sci. Lett.* **519**, 109–119 (2019).
12. Fuchs, S., Schütz, F., Förster, H. J. & Förster, A. Evaluation of common mixing models for calculating bulk thermal conductivity of sedimentary rocks: Correction charts and new conversion equations. *Geothermics* **47**, 40–52 (2013).
13. Chang, Y.-Y., Hsieh, W.-P., Tan, E. & Chen, J. Hydration-reduced lattice thermal conductivity of olivine in Earth ' s upper mantle. *Proc. Natl. Acad. Sci. USA* **114**, 4078 (2017).
14. Hsieh, W.-P. *et al.* Low Thermal Conductivity of Hydrous Phase D Leads to a Self-Preservation Effect Within a Subducting Slab. *J. Geophys. Res. Solid Earth* **127**, e2022JB024556 (2022).
15. Hsieh, W. P., Marzotto, E., Tsao, Y. C., Okuchi, T. & Lin, J. F. High thermal conductivity of stishovite promotes rapid warming of a sinking slab in Earth's mantle. *Earth Planet. Sci. Lett.* **584**, 117477 (2022).
16. van Keken, P. E., Hacker, B. R., Syracuse, E. M. & Abers, G. A. Subduction factory: 4. Depth-dependent flux of H2O from subducting slabs worldwide. *J. Geophys. Res. Solid Earth* **116**, B01401 (2011).
17. John, T., Scambelluri, M., Frische, M., Barnes, J. D. & Bach, W. Dehydration of subducting serpentinite: Implications for halogen mobility in subduction zones and the deep halogen cycle. *Earth Planet. Sci. Lett.* **308**, 65–76 (2011).
18. Faccenda, M., Gerya, T. V., Mancktelow, N. S. & Moresi, L. Fluid flow during slab unbending and dehydration: Implications for intermediate-depth seismicity, slab weakening and deep water recycling. *Geochemistry, Geophys. Geosystems* **13**, Q01010 (2012).
19. Davy, R. G. *et al.* Wide-Angle Seismic Imaging of Two Modes of Crustal Accretion in Mature Atlantic Ocean Crust. *J. Geophys. Res. Solid Earth* **125**, e2019JB019100 (2020).
20. Su, C., Liu, Y., Song, W., Fan, D. & Wang, Z. Thermodynamic properties of San Carlos olivine at high temperature and high pressure. *Acta Geochim.* **37**, 171–179 (2018).
21. Hilairet, N., Daniel, I. & Reynard, B. Equation of state of antigorite , stability field of serpentines , and seismicity in subduction zones. *Geophys. Res. Lett.* **33**, L02302 (2006).
22. Osako, M., Yoneda, A. & Ito, E. Thermal diffusivity, thermal conductivity and heat capacity of serpentine (antigorite) under high pressure. *Phys. Earth Planet. Inter.* **183**, 229–233 (2010).
23. Padrón-Navarta, J. A., Tommasi, A., Garrido, C. J. & López Sánchez-Vizcaíno, V. Plastic deformation and development of antigorite crystal preferred orientation in high-pressure serpentinites. *Earth Planet. Sci. Lett.* **349**–**350**, 75–86 (2012).
24. Nishii, A., Wallis, S. R., Mizukami, T. & Michibayashi, K. Subduction related antigorite CPO patterns from forearc mantle in the Sanbagawa belt, southwest Japan. *J. Struct. Geol.* **33**, 1436–1445 (2011).
25. Jung, H. Seismic anisotropy produced by serpentine in mantle wedge. *Earth Planet. Sci. Lett.* **307**, 535–543 (2011).
26. Satta, N. *et al.* Single-Crystal Elasticity of Antigorite at High Pressures and Seismic Detection of Serpentinized Slabs. *Geophys. Res. Lett.* **49**, e2022GL099411 (2022).
27. Allen, R. W., Collier, J. S. & Henstock, T. J. The Role of Crustal Accretion Variations in Determining Slab Hydration at an Atlantic Subduction Zone. *J. Geophys. Res. Solid Earth* **127**, e2022JB024349 (2022).
28. Cooper, G. F. *et al.* Variable water input controls evolution of the Lesser Antilles

volcanic arc. *Nature* **582**, 525–529 (2020).
29. Kaminski, É., Ribe, N. M. & Browaeys, J. T. D-Rex, a program for calculation of seismic anisotropy due to crystal lattice prefferred orientation in the convective upper mantle. *Geophys. J. Int.* **158**, 744–752 (2004).
30. Klemens, P. G. Phonon Scattering and Thermal Resistance Due to Grain Boundaries ~. *Int. J. Thermophys.* **15**, 1345–1351 (1994).
31. Powell, R. W. & Griffiths, E. The Variation with Temperature of the Thermal Con ductivity and the X-Ray Structure of some Micas I — The Thermal Conductivity up to 600C. *Proc. Roy. Soc. London. Ser. A-Math. Phys. Sci.Phys. Sci.* **163**, 189–198 (1937).
32. Wood, W. The Variation with Temperature of the Thermal Conductivity and the X-ray Structure of some Micas II-The X-ray Examination of the Structure. *Proc. Roy. Soc. London. Ser. A-Math. Phys. Sci.Phys. Sci.* **163**, 199 (1937).
33. Zheng, X., Cahill, D. G., Krasnochtchekov, P., Averback, R. S. & Zhao, J. C. High-throughput thermal conductivity measurements of nickel solid solutions and the applicability of the Wiedemann-Franz law. *Acta Mater.* **55**, 5177–5185 (2007).
34. Cahill, D. G. & Watanabe, F. Thermal conductivity of isotopically pure and Ge-doped Si epitaxial layers from 300 to 550 K. *Phys. Rev. B* **70**, 235322 (2004).
